# Association Between Oral Frailty and Body Mass Index in Late-Stage Older Adults Stratified by Sarcopenia Status

**DOI:** 10.3390/nu18172835

**Published:** 2026-08-28

**Authors:** Masaki Hayase, Kazuhito Miura, Kimiya Ozaki, Yasushi Tamada, Maki Shirobe, Keiko Motokawa, Misato Hayakawa, Hirohiko Hirano, Takahiro Ono, Eri Arai, Masanori Iwasaki, Akira Adachi, Takao Watanabe, Shigekazu Komoto, Yutaka Watanabe

**Affiliations:** 1 Gerodontology, Department of Oral Health Science, Faculty of Dental Medicine, Hokkaido University, Sapporo 060-8586, Japan; hayasemasaki@den.hokudai.ac.jp (M.H.); kmiura@den.hokudai.ac.jp (K.M.); o.kimiya75@gmail.com (K.O.); tamada@den.hokudai.ac.jp (Y.T.); 2 Research Team for Promoting Independence and Mental Health, Tokyo Metropolitan Institute for Geriatrics and Gerontology, Tokyo 173-0015, Japan; mshirobe@tmig.or.jp (M.S.); kmoto@tmig.or.jp (K.M.); erurinrun0424@gmail.com (M.H.); h-hiro@gd5.so-net.ne.jp (H.H.); 3 Department of Geriatric Dentistry, Osaka Dental University, Hirakata 573-1121, Japan; ono-t@cc.osaka-dent.ac.jp; 4 Department of Dentistry, Hokuto Hospital, Obihiro 080-0833, Japan; ae.hok9den26.obi5@gmail.com; 5 Division of Dental Public Health, Department of Oral Health Science, Graduate School of Dental Medicine, Hokkaido University, Sapporo 060-8586, Japan; iwasaki@den.hokudai.ac.jp; 6 Tottori Dental Association, Tottori 680-0841, Japan; ya.adachi@gmail.com (A.A.); renkei@ttrda.jp (T.W.); 7 Department of Health Care Policy, Faculty of Medicine and Graduate School of Medicine, Hokkaido University, Sapporo 060-8638, Japan; komoto@med.hokudai.ac.jp

**Keywords:** late-stage older adults, obesity, oral frailty, sarcopenia

## Abstract

Objectives: Oral frailty is associated with sarcopenia and underweight; however, its relationship with sarcopenic obesity remains unclear. This cross-sectional study investigated the associations between oral frailty and body mass index (BMI) categories (underweight/obesity), stratified by sarcopenia status. Methods: Data were obtained from dental checkups for 3829 older adults in Tottori, Japan (1489 men and 2340 women, mean age: 80.1 ± 4.3 years). Participants were divided into sarcopenia risk and non-sarcopenia groups by using the Yubi–Wakka test and then further subdivided by BMI into underweight, normal weight, or obese. Oral frailty was defined as meeting three or more of six oral frailty assessment criteria adapted from previous studies. Multinomial logistic regression analysis was performed to examine the association between oral frailty and each BMI category, with normal weight used as the reference category. Results: In the sarcopenia risk group, both obesity and underweight were significantly associated with oral frailty. The odds ratio (OR) for obesity was 1.727, with a 95% confidence interval (CI) of 1.092–2.732, and the OR for underweight was 1.443, with a 95% CI of 1.066–1.952. In contrast, no significant association between BMI category and oral frailty was observed in the non-sarcopenia group. Conclusions: Among late-stage older adults at risk of sarcopenia, oral frailty was associated with both obesity and underweight, with different contributing factors identified for each BMI category.

## 1. Introduction

Japan’s aging rate, defined as the proportion of the population aged 65 years or older, reached 29.3% in 2024, the highest level worldwide. Similarly, late-stage older adults, defined as those aged ≥75 years, currently account for 16.8% of the total population [1]. As the number of late-stage older adults increases, the proportion of individuals requiring medical and long-term care also rises, highlighting the need for measures and interventions to maintain and improve functional capacity and prevent long-term care dependency.

Preventing long-term care dependency among older adults requires early and appropriate intervention for frailty, which is characterized by physical and mental decline [2]. Sarcopenia is a key component of the frailty cycle and is considered to trigger a negative cycle involving undernutrition, low body weight, reduced physical activity, and decreased basal metabolism [3]. Although sarcopenia frequently coexists with underweight, known as sarcopenic underweight, increasing attention has recently been paid to the coexistence of sarcopenia and obesity, known as sarcopenic obesity [4]. Sarcopenic obesity is driven by several factors, including unhealthy dietary habits and physical inactivity [5]. Oral health has also raised concern in this context. Studies have reported associations between sarcopenic obesity and impaired oral status [6], including decreased tongue pressure [7].

Oral frailty has also gained attention as a factor associated with frailty. Oral frailty is defined as the accumulation of slight declines in oral function, including tooth loss and difficulties in eating and communication, which increase the risk of impaired oral functional capacity; however, appropriate intervention and treatment can effectively improve this condition [8]. Oral frailty is thought to contribute to sarcopenia and malnutrition, particularly underweight. Furthermore, while an association with increased risk of weight gain is suggested due to the link between poor oral function and overnutrition [9], the direct relationship between oral frailty and obesity has not been sufficiently examined. Sarcopenic obesity and sarcopenic underweight are considered clinically distinct conditions, despite sharing similar pathophysiological mechanisms [5]. Based on these findings, we hypothesized that obesity and underweight may have different associations with oral frailty and that different approaches may be required for each condition.

Therefore, this cross-sectional study investigated the association of oral frailty with underweight and obesity, stratified by sarcopenia status, and compared differences in these associations.

## 2. Materials and Methods

### 2.1. Setting and Participants

This study targeted insured individuals aged ≥75 years who lived in Tottori Prefecture, Japan, and were covered by the medical care system for older adults. The study period was 6 years, from April 2016 to March 2022.

To promote participation in dental checkups for late-stage older adults, flyers announcing the checkups were mailed together with health insurance certificates for the medical care system. Posters were also displayed at community centers, meeting halls, public health centers, and dental clinics, and public information channels in each municipality were used to disseminate information. Dental checkups for late-stage older adults are categorized as a voluntary duty in Japan. Therefore, participation in this study was voluntary, and participants responded to the study announcement on their own initiative.

### 2.2. Research Methods

This study was conducted at 163 dental clinics. To standardize the assessment criteria, the examining dentists received prior training on the research methods, recording procedures, and judgment criteria. The checkup results were sent to the prefectural insurer and compiled into a database. In addition, anonymized claims data for the participants were provided by the prefectural insurer and integrated with the dental checkup data. The use of claims data for research purposes was announced using an opt-out method, with information posted on the website of the prefectural dental association and at the dental clinics where the checkups were performed.

### 2.3. Research Items

#### 2.3.1. Medical Questionnaire

Before the dental checkup, the participants completed a self-administered 22-item questionnaire that included items on age, sex, height, weight, number of medications per day, the Simplified Nutritional Appetite Questionnaire (SNAQ), and the dietary variety score (DVS). The questionnaire also included the Yubi–Wakka test [10], a self-screening assessment for sarcopenia. These responses were verified by the examining dentists during the dental checkup.

#### 2.3.2. Oral Assessment

The number of current teeth, tongue and lip motor function, and masticatory function were assessed [11].

#### 2.3.3. Systemic Diseases Evaluation

Systemic diseases were evaluated using the Charlson Comorbidity Index [12] and diagnostic information from claims data coded according to the International Classification of Diseases, 10th revision [13]. Nineteen comorbidities were scored, and the total score was calculated.

### 2.4. Measurement Methods

#### 2.4.1. Body Mass Index

Body mass index (BMI) was calculated using participants’ height and weight. Participants were classified by BMI as underweight (BMI < 18.5) or normal weight (BMI 18.5–24.9) or obese (BMI ≥ 25), according to the Japan Society for the Study of Obesity Guidelines for the Management of Obesity [14].

#### 2.4.2. Yubi–Wakka Test

The Yubi–Wakka test [10] is a self-screening assessment tool and was used to classify participants according to sarcopenia risk. The assessment was performed in a sitting position by forming a ring with the thumb and index finger around the calf circumference. The results were classified into three categories: “bigger,” “just fit,” and “smaller.” Participants categorized as “smaller” were classified as being at risk of sarcopenia and were assigned to the sarcopenia risk group.

#### 2.4.3. Nutritional Indicators

The SNAQ [15] and DVS [16] were used as nutritional indicators. The SNAQ comprises four questions on appetite, satiety, taste perception, and the number of meals eaten, with a total score of 20 points. The DVS assesses the weekly intake frequency of 10 food groups: meat, fish and shellfish, milk, seaweed, soybean products, fruits, eggs, green and yellow vegetables, oils and fats, and potatoes. The total score is 10 points.

#### 2.4.4. Oral Frailty

Oral frailty was assessed based on Arai et al. [17] and Nakagawa et al. [18] as follows:

From the medical questionnaire, “Has the difficulty in consuming hard food increased compared to 6 months ago?”: Yes.

From the medical questionnaire, “Do you experience choking when consuming tea or soup?”: Yes.

Number of current teeth: fewer than 20 teeth, excluding root remnants.

Oral diadochokinesis:/pa/less than 5.2 times/s in men and less than 5.6 times/s in women.

Oral diadochokinesis:/ka/less than 4.4 times/s in men and less than 5.0 times/s in women.

Masticatory performance test: a score of 0, 1, or 2 after chewing gummy jelly.

Oral diadochokinesis [19] was assessed using an oral function measuring device (Kenkoukun Handy; Takei Scientific Instruments, Niigata, Japan). Participants were instructed to pronounce the evaluation syllables as rapidly and continuously as possible, and the number of repetitions per second was recorded. The masticatory performance test [11] was conducted using a test gummy jelly measuring approximately 2 cm × 2 cm × 1 cm, provided by UHA Mikakuto, Osaka, Japan. After 30 mastication cycles, the gummy jelly fragments were compared with visual scales for assessment. Oral frailty was considered to meet ≥3 of these assessment criteria.

### 2.5. Analysis Method

Participants were divided into sarcopenia risk and non-sarcopenia groups by using the Yubi–Wakka test and then further subdivided by BMI into underweight, normal weight, or obese. Intragroup comparisons and multinomial logistic regression analyses were performed within each group. Because dental clinics typically lack body composition analyzers and handgrip dynamometers, we used the Yubi–Wakka test, which is positioned as a screening tool in the Asian Working Group for Sarcopenia 2025 [4]. In cross-sectional analyses, even after adjustment for covariates including calf pedal edema, the Yubi–Wakka test remained significantly associated with a high prevalence of pre-sarcopenia and sarcopenia [10].

Intragroup comparisons included age, sex, number of medications per day, SNAQ, DVS, Charlson Comorbidity Index, and oral frailty assessment items. For continuous variables, the Kruskal–Wallis test was used for age, number of medications per day, DVS, and Charlson Comorbidity Index, and one-way analysis of variance was used for SNAQ. The normality of data distribution was visually assessed using histograms. Categorical variables, including sex and oral frailty assessment items, were analyzed using the χ^2^ test.

Multinomial logistic regression analysis was used to examine the association between oral frailty and each BMI category within each group, with normal weight used as the reference category. Models were adjusted a priori for age, sex, number of medications per day, SNAQ [15], DVS [18], and Charlson Comorbidity Index [12] based on their clinical relevance and previously reported associations. All variables included in the multinomial logistic regression model had variance inflation factor values of <5, indicating no multicollinearity, as shown in the Supplementary Materials (Table S1). All analyses were performed using SPSS version 29.0 (IBM Corp., Armonk, NY, USA), and statistical significance was set at *p* < 0.05.

## 3. Results

### 3.1. Classification of Participants

As of 2019, approximately 90,000 late-stage older adults lived in Tottori Prefecture, Japan [20]. In this study, only data from the initial checkup were analyzed. During the study period from April 2016 to March 2022, 4681 late-stage older adults underwent dental checkups. Of these, we included 3829 individuals (i.e., approximately 4% of 90,000), including 1489 men and 2340 women, with a mean age of 80.1 ± 4.3 years. We excluded 852 of 4681 individuals with incomplete data: those whose BMI could not be calculated (*n* = 640), those with no results for the Yubi–Wakka test (*n* = 82), those whose recorded age was under 75 years (*n* = 31), and those with incomplete items in the oral frailty assessment (*n* = 99).

**Figure 1:**
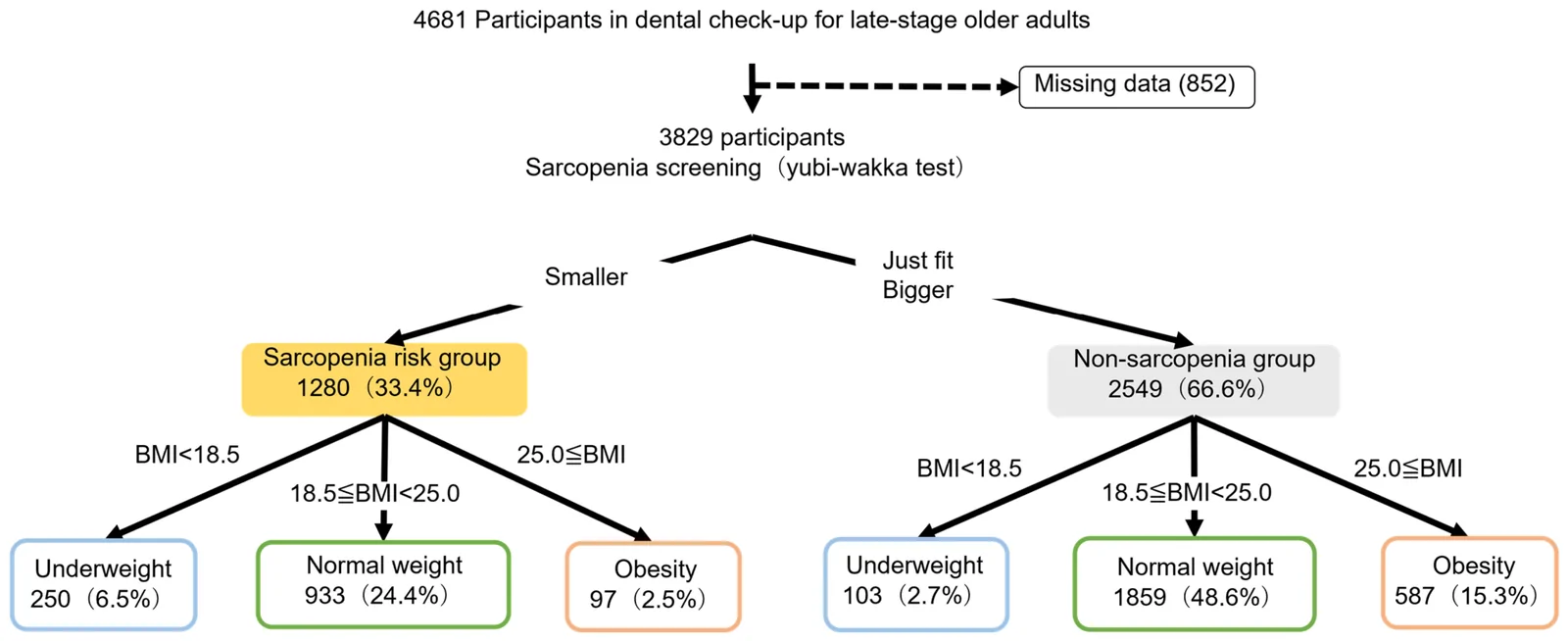
Flowchart of the study participant selection process.

Among the participants, 1280 individuals (33.4%) were classified into the sarcopenia risk group. Within this group, 250 individuals (6.5%) were underweight, 933 (24.4%) had normal weight, and 97 (2.5%) had obesity. The remaining 2549 individuals (66.6%) were classified into the non-sarcopenia group. Within this group, 103 individuals (2.7%) were underweight, 1859 (48.6%) had normal weight, and 587 (15.3%) had obesity (Figure 1).

### 3.2. Basic Characteristics of Participants and Oral Frailty Determination

Overall, 47.7% of participants (1825 individuals) were categorized as having oral frailty. Comparisons between the sarcopenia risk and non-sarcopenia groups showed considerable differences in age, SNAQ, DVS, and the oral frailty assessment item “Has the difficulty in consuming hard food increased compared to 6 months ago?” (Table 1).

In the sarcopenia risk group, 49.0% of participants (627 individuals) were categorized as having oral frailty. Intragroup comparisons showed considerable differences in age and sex, as well as the oral frailty assessment items “Has the difficulty in consuming hard food increased compared to 6 months ago?” and oral diadochokinesis of/ka/. Oral frailty determination also differed considerably among body mass index categories in this group (Table 2).

In the non-sarcopenia group, 47.0% of participants (1198 individuals) were categorized as having oral frailty. Intragroup comparisons showed considerable differences in age, sex, number of medications per day, and SNAQ, as well as the oral frailty assessment items of number of current teeth and masticatory performance test (Table 3).

We performed these exploratory comparisons and relied primarily on multivariable regression models for inference, which is why multiple comparisons were not conducted.

### 3.3. Association of Oral Frailty with Underweight and Obesity in Each Group

Multinomial logistic regression analysis was performed to examine the association of oral frailty with underweight and obesity in the sarcopenia risk (likelihood ratio [LR] χ^2^ (14) = 38.07, *p* < 0.001, McFadden pseudo-R^2^ = 0.021) and non-sarcopenia (LR χ^2^ (14) = 85.08, *p* < 0.001, McFadden pseudo-R^2^ = 0.025) groups (Table 4). The crude estimates are attached to the Supplementary Materials (Table S2). In the sarcopenia risk group, both obesity (odds ratio [OR], 1.727; 95% confidence interval [CI], 1.092–2.732) and underweight (OR, 1.443; 95% CI, 1.066–1.952) were significantly associated with oral frailty. No significant association of oral frailty with underweight or obesity was observed in the non-sarcopenia group.

Associated factors also differed within the sarcopenia risk group. Obesity was associated with sex, with women having lower odds of obesity (OR: 0.569, 95% CI: 0.361–0.895), whereas underweight was associated with the DVS (OR: 1.081, 95% CI: 1.015–1.152) and the Charlson Comorbidity Index (OR: 1.190, 95% CI: 1.006–1.406).

## 4. Discussion

To the best of our knowledge, this is the first study to demonstrate an association between obesity and oral frailty in older individuals at risk of sarcopenia. In addition, the associated factors differed between the obesity and underweight groups. Because oral frailty is thought to eventually lead to frailty, sarcopenia, and malnutrition [8], our finding that oral frailty was associated only with the sarcopenia risk group supports the results of a previous study [21]. Furthermore, the finding that factors other than oral frailty differed between the obesity and underweight groups within the sarcopenia risk group suggests the need for measures tailored to each factor while also addressing oral frailty.

In the obesity at risk of sarcopenia group, male sex was associated with obesity compared with the normal weight group. One possible explanation is the influence of age-related declines in testosterone levels. Testosterone has various effects on the male body, including promoting muscle protein synthesis and lipolysis. Loss of these functions due to decreased testosterone may lead to reduced basal metabolism caused by decreased muscle mass, promote visceral fat accumulation, and ultimately lead to obesity [22]. Sarcopenia and obesity share several common factors, including lifestyle habits, aging, and endocrine changes, and can create a vicious cycle by exerting synergistic effects on each other. Because the coexistence of sarcopenia and obesity increases the risk of lifestyle-related disease complications and raises mortality rates compared with obesity alone [5], early improvement is necessary. Based on these findings, interventions aimed at maintaining muscle strength and testosterone secretion alongside addressing oral frailty may represent a hypothetical approach. While the present study was cross-sectional and did not directly evaluate training effects, resistance exercise, which involves repetitive movements that place a load on the muscles, is considered useful for increasing testosterone secretion. Although this study targeted late-stage older adults, resistance exercise has been reported to increase muscle mass and strength in older individuals [23,24] and to increase testosterone levels [25]. Resistance training may also benefit oral function; chin-tuck and tongue exercises were associated with tongue pressure and swallowing pressure in older adults and showed a trend toward improved oral intake [26]. Therefore, resistance training for both the whole body and oral cavity warrants further investigation as potential therapeutic strategies for men with obesity at risk of sarcopenia.

In the underweight at risk of sarcopenia group, higher DVSs and the Charlson Comorbidity Index were associated with underweight compared with the normal weight. Although a correlation between decreased DVSs and oral frailty has been reported [27], our results differed from this previous finding. A high DVS is generally considered to reflect consumption of a nutrient-dense diet and to help suppress the decline in physical function [28]. However, a higher DVS may be associated with lower BMI and lower body fat [29]. This previous study [29] pointed to the low energy density of overall diets. In addition, a higher DVS has been reported to be associated with increased intake of low-energy foods, such as vegetables and fruits [16]. In the present study, low body weight may have occurred because of the low energy density of the diet, although this hypothesis requires further verification. Regarding the association with the Charlson Comorbidity Index, many chronic and severe diseases can cause gastrointestinal symptoms or metabolic abnormalities and lead to decreased appetite and impaired digestion or absorption of nutrients, thereby increasing susceptibility to malnutrition [30]. These findings suggest that in addition to addressing oral frailty, interventions for the underweight at-risk-of-sarcopenia group may need to emphasize an energy-dense diet and nutrient absorption. From a clinical perspective, rather than providing uniform dietary guidance, individualized assessment of oral, swallowing, and nutritional status is essential. Masticatory load could then be optimized to increase the number of chews according to individual function, such as by selecting foods with harder textures or using cooking methods that retain a chewy consistency in line with the Dietary Reference Intakes for Japanese [31]. Consuming foods with a firm texture increases the masticatory load, leading to increased masticatory muscle activity [32,33]. Furthermore, increasing the duration of taste stimulation and mastication has been reported to increase blood flow in the celiac artery and enhance upper gastrointestinal activity [34]. Based on these reports, the efficiency of nutrient absorption may also be improved. Therefore, nutritional interventions combined with oral frailty management warrant further investigation as a therapeutic strategy for individuals who are underweight at risk of sarcopenia.

In the non-sarcopenia group, no association with oral frailty was observed. This may be because systemic muscle strength, including that of the oral cavity, was maintained due to the absence of sarcopenia. In the non-sarcopenic obesity group, obesity was associated with the Charlson Comorbidity Index, number of medications per day, and SNAQ, which is consistent with previous studies [35,36]. In the non-sarcopenic underweight group, underweight was associated with age and female sex, similar to the results of the National Health and Nutrition Survey in Japan [37]. These findings suggest that the non-sarcopenia group included individuals with obesity or underweight due to general physiological factors.

Oral frailty in this study was determined per Arai et al. [17] and Nakagawa et al. [18]; however, these criteria differ slightly from those used in the original study by Tanaka et al. [21] because tests requiring specialized equipment, such as tongue pressure measurement, could not be performed. Therefore, the number of individuals identified as having oral frailty may have been higher than that identified using the original definition, and caution is required when interpreting the evaluation criteria. The Yubi–Wakka test was used to assess sarcopenia, and BMI was used to assess obesity and underweight. These are practical and useful screening evaluation items in the diagnostic algorithm for sarcopenic obesity in Japan [4]. The Yubi–Wakka test is a self-screening method whose validity for identifying sarcopenia has been reported [10]. BMI is also used for obesity classification in the Japanese Guidelines for the Management of Obesity [14]. Because this was a large-scale survey, grouping was conducted using these methods with an emphasis on simplicity. However, future studies should examine these associations using the Global Leadership Initiative on Malnutrition criteria [38] and international criteria for sarcopenic obesity [39].

Compared with previous studies [18], this study includes 943 participants whose analysis data overlap, along with 2886 newly added participants, out of a total of 3829. This is due to differences in participant selection methods [18], as well as the extension of the observation period and the expansion of the sample size in this study. In this study, 33.4% of participants were identified as having sarcopenia risk through the Yubi–Wakka test, which is a higher proportion than that reported previously [10,40]. Although studies have included individuals aged ≥65 years, we included late-stage older adults aged ≥75 years, which may have had a higher proportion of individuals with marked physiological and physical decline. Regarding the proportions of obesity and underweight, no differences were observed compared with a previous study investigating the distribution of sarcopenia and BMI [41]. However, among participants at risk of sarcopenia, the obesity group had a smaller sample size than the normal-weight group, which may reduce the precision of the estimates and increase the uncertainty of the reported odds ratios. Oral frailty was examined by adding cases to those in previous studies [17,18], but the trends remained unchanged. Regarding DVS and SNAQ, compared with previous studies with a high proportion of late-stage older adults (aged ≥ 75 years), DVSs were higher [42], whereas SNAQ scores did not differ from those in a previous study [15]. Based on the above, the participants in this study are considered generally representative of community-dwelling older adults in Japan.

This study has some limitations. First, its cross-sectional design precluded the determination of the causal relationship between oral frailty and the coexistence of sarcopenia risk with obesity or underweight. Furthermore, residual confounding, such as from socioeconomic status and lifestyle factors, cannot be ruled out. In particular, variables such as educational level, physical activity, and denture use may have influenced the observed associations. Second, the study was conducted in a single prefecture in Japan, and the number of included participants was very small, accounting for only over 4% of late-stage older adults in the prefecture. Additionally, participation was voluntary, and it is possible that more individuals with a high interest in their health or those experiencing health issues participated. Thus, sampling or selection bias may have been introduced. Third, the assessment of sarcopenia and BMI had limitations. Sarcopenia was assessed using only the Yubi–Wakka test, which is a screening method and not a definitive diagnosis. Therefore, the possible influence of subcutaneous fat in the lower leg, edema, and circulatory failure caused by obesity on the Yubi–Wakka test results could not be excluded. BMI was calculated based on a self-administered questionnaire, and there may have been discrepancies between the calculated and measured values. Future studies should obtain data sufficient for a definitive diagnosis of sarcopenia and employ longitudinal designs to clarify the temporal associations between oral frailty and the coexistence of sarcopenia with obesity or underweight.

## 5. Conclusions

This study revealed that oral frailty was associated with both obesity and underweight among late-stage older adults classified with sarcopenia risk. In addition, this study further identified differences in the factors associated with obesity and underweight. These findings suggest the potential need to implement distinct strategies tailored to each group, in addition to addressing oral frailty. Moving forward, it is necessary to confirm through interventional studies whether oral frailty countermeasures are effective for sarcopenic obesity and sarcopenic underweight and to establish specific approaches for systemic management that incorporate oral health.

## Figures and Tables

**Table 1:** Comparison of participant characteristics and oral frailty assessment items between the sarcopenia risk and non-sarcopenia groups.

	Overall (*n* = 3829)	Sarcopenia Risk Group (*n* = 1280)	Non-Sarcopenia Group (*n* = 2549)	*p*-Value
Characteristics				
Age (years)	79 (77–83)	80 (77–84)	79 (76–82)	<0.001 ^a^
Sex (female)	2340 (61.1)	765 (59.8)	1575 (61.8)	0.226 ^b^
Number of medications per day	4 (2–5)	4 (2–5)	3 (2–5)	0.476 ^a^
Simplified Nutritional Appetite Questionnaire (Mean ± SD)	14.7 ± 1.7	14.6 ± 1.7	14.8 ± 1.6	0.019 ^c^
Dietary variety score	8 (6–9)	8 (6–9)	8 (6–9)	0.004 ^a^
Charlson Comorbidity Index	0 (0–1)	0 (0–1)	0 (0–1)	0.300 ^a^
Oral frailty assessment items				
Has the difficulty in consuming hard food increased compared to 6 months ago? (Yes)	1224 (32.0)	440 (34.3)	784 (30.8)	0.024 ^b^
Do you experience choking when consuming tea or soup? (Yes)	921 (24.1)	307 (24.0)	614 (24.1)	0.944 ^b^
Number of current teeth (<20)	1850 (48.3)	642 (50.2)	1208 (47.4)	0.106 ^b^
ODK/pa/(Male < 5.2 times/s, Female < 5.6 times/s)	2451 (64.0)	827 (64.6)	1624 (63.7)	0.585 ^b^
ODK/ka/(Male < 4.4 times/s, Female < 5.0 times/s)	1928 (50.4)	654 (51.1)	1274 (50.0)	0.516 ^b^
Masticatory performance test (Scores of 0, 1, or 2)	1133 (29.6)	392 (30.6)	741 (29.1)	0.320 ^b^
Oral frailty assessment results (3 items or more)	1825 (47.7)	627 (49.0)	1198 (47.0)	0.246 ^b^

Notes: Data are presented as *n* (%) or median (interquartile range) unless otherwise indicated. Abbreviations: ODK, Oral diadochokinesis; SD, standard deviation. ^a^ Mann–Whitney U-test; ^b^ χ^2^ test; ^c^ *t* test.

**Table 2:** Intragroup comparison of participant characteristics and oral frailty assessment items within the sarcopenia risk group.

	Normal Weight (*n* = 933)	Obesity (*n* = 97)	Underweight (*n* = 250)	*p*-Value
Characteristics				
Age (years)	80 (77–84)	80 (76–83)	80 (77–85)	0.049 ^a^
Sex (female)	555 (59.5)	47 (48.5)	164 (65.2)	0.016 ^b^
Number of medications per day	3 (2–5)	4 (2–5)	4 (2–6)	0.331 ^a^
Simplified Nutritional Appetite Questionnaire (Mean ± SD)	14.6 ± 1.7	14.7 ± 1.5	14.4 ± 2.0	0.078 ^c^
Dietary variety score	8 (5–9)	8 (6–9)	8 (6–9)	0.225 ^a^
Charlson Comorbidity Index	0 (0–1)	0 (0–1)	0 (0–1)	0.206 ^a^
Oral frailty assessment items				
Has the difficulty in consuming hard food increased compared to 6 months ago? (Yes)	296 (31.7)	35 (36.1)	109 (43.6)	0.002 ^b^
Do you experience choking when consuming tea or soup? (Yes)	214 (22.9)	23 (23.7)	70 (28.0)	0.249 ^b^
Number of current teeth (<20)	465 (49.8)	52 (53.6)	125 (50.0)	0.778 ^b^
ODK/pa/(Male < 5.2 times/s, Female < 5.6 times/s)	592 (63.5)	62 (63.9)	173 (69.2)	0.238 ^b^
ODK/ka/(Male < 4.4 times/s, Female < 5.0 times/s)	466 (49.9)	42 (43.3)	146 (58.4)	0.017 ^b^
Masticatory performance test (Scores of 0, 1, or 2)	277 (29.7)	30 (30.9)	85 (34.0)	0.421 ^b^
Oral frailty assessment results (3 items or more)	434 (46.5)	52 (53.6)	141 (56.4)	0.014 ^b^

Notes: Data are presented as *n* (%) or median (interquartile range) unless otherwise indicated. Abbreviations: ODK, Oral diadochokinesis; SD, standard deviation. ^a^ Kruskal–Wallis test; ^b^ χ^2^ test; ^c^ One-way analysis of variance (ANOVA).

**Table 3:** Intragroup comparison of participant characteristics and oral frailty assessment items within the non-sarcopenia group.

	Normal Weight (*n* = 1859)	Obesity (*n* = 587)	Underweight (*n* = 103)	*p*-Value
Characteristics				
Age (years)	79 (76–82)	79 (76–82)	81 (77–84)	0.026 ^a^
Sex (female)	1135 (61.1)	357 (60.8)	83 (80.6)	<0.001 ^b^
Number of medications per day	3 (2–5)	4 (2–6)	3 (1–5)	<0.001 ^a^
Simplified Nutritional Appetite Questionnaire (Mean ± SD)	14.8 ± 1.6	14.9 ± 1.7	14.7 ± 1.7	0.049 ^c^
Dietary variety score	8 (6–9)	8 (6–10)	8 (7–10)	0.387 ^a^
Charlson Comorbidity Index	0 (0–1)	0 (0–0)	0 (0–1)	0.153 ^a^
Oral frailty assessment items				
Has the difficulty in consuming hard food increased compared to 6 months ago? (Yes)	561 (30.2)	189 (32.2)	34 (33.0)	0.574 ^b^
Do you experience choking when consuming tea or soup? (Yes)	441 (23.7)	147 (25.0)	26 (25.2)	0.777 ^b^
Number of current teeth (<20)	853 (45.9)	304 (51.8)	51 (49.5)	0.040 ^b^
ODK/pa/(Male < 5.2 times/s, Female < 5.6 times/s)	1195 (64.3)	360 (61.3)	69 (67.0)	0.336 ^b^
ODK/ka/(Male < 4.4 times/s, Female < 5.0 times/s)	932 (50.1)	288 (49.0)	54 (52.4)	0.794 ^b^
Masticatory performance test (Scores of 0, 1, or 2)	505 (27.1)	202 (34.4)	34 (33.0)	0.002 ^b^
Oral frailty assessment results (3 items or more)	853 (45.9)	294 (50.1)	51 (49.5)	0.180 ^b^

Notes: Data are presented as *n* (%) or median (interquartile range) unless otherwise indicated. Abbreviations: ODK, Oral diadochokinesis; SD, standard deviation. ^a^ Kruskal–Wallis test; ^b^ χ^2^ test; ^c^ one-way ANOVA.

**Table 4:** Results of the multinomial logistic regression analysis of factors associated with underweight and obesity.

	Sarcopenia Risk Group				Non-Sarcopenia Group			
	Normal Weight (Reference)				Normal Weight (Reference)			
	Underweight		Obesity		Underweight		Obesity	
	OR (95% CI)	*p*-Value	OR (95% CI)	*p*-Value	OR (95% CI)	*p*-Value	OR (95% CI)	*p*-Value
Age (years)	1.020 (0.987–1.054)	0.230	0.969 (0.919–1.021)	0.233	1.087 (1.039–1.138)	<0.001	0.987 (0.963–1.011)	0.298
Number of medications per day	1.017 (0.967–1.071)	0.510	1.037 (0.963–1.117)	0.337	0.961 (0.888–1.041)	0.329	1.109 (1.073–1.145)	<0.001
Simplified Nutritional Appetite Questionnaire	0.938 (0.862–1.020)	0.135	1.010 (0.883–1.155)	0.883	0.936 (0.823–1.063)	0.309	1.111 (1.041–1.184)	0.001
Dietary variety score	1.081 (1.015–1.152)	0.015	1.085 (0.986–1.194)	0.096	1.011 (0.919–1.113)	0.819	0.996 (0.954–1.040)	0.861
Charlson Comorbidity Index	1.190 (1.006–1.406)	0.042	0.995 (0.764–1.295)	0.968	1.096 (0.856–1.403)	0.466	0.846 (0.748–0.957)	0.008
Sex: male	(reference)		(reference)		(reference)		(reference)	
female	1.179 (0.864–1.609)	0.300	0.569 (0.361–0.895)	0.015	2.658 (1.567–4.510)	<0.001	0.934 (0.760–1.149)	0.521
Oral frailty assessment results, not applicable	(reference)		(reference)		(reference)		(reference)	
applicable	1.443 (1.066–1.952)	0.018	1.727 (1.092–2.732)	0.019	0.855 (0.554–1.320)	0.479	1.150 (0.939–1.410)	0.177
McFadden pseudo-R^2^	0.021				0.025			
LR χ^2^	38.07 *				85.08 *			

Abbreviations: CI, confidence interval; OR, odds ratio; LR, likelihood ratio. * *p* < 0.001.

## Data Availability

The data that support the findings of this study are available from the Tottori Dental Association. Restrictions apply to the availability of these data, which were used under license for this study. Therefore, the data are available from the authors with permission from the Tottori Dental Association.

## Supplementary Materials

The following supporting information can be downloaded at https://www.mdpi.com/article/10.3390/nu18172835/s1. Table S1: Multicollinearity statistics; Table S2: Crude estimates from multinomial logistic regression analysis of factors associated with underweight and obesity.

## Abbreviations

The following abbreviations are used in this manuscript:

BMI	body mass index
CI	confidence interval
DVS	dietary variety score
LR	likelihood ratio
ODK	oral diadochokinesis
OR	odds ratio
SD	standard deviation
SNAQ	Simplified Nutritional Appetite Questionnaire

## References

[1] Cabinet Office Japan Annual Report on the Ageing Society. Summary FY2025. https://www8.cao.go.jp/kourei/english/annualreport/2025/pdf/2025.pdf.

[2] Matsuo R., Matsumoto N., Mitsuhashi T., Takao S., Yorifuji T. (2023). Frailty and all-cause and cause-specific mortality in Japan. Arch. Gerontol. Geriatr..

[3] Xue Q.L., Bandeen-Roche K., Varadhan R., Zhou J., Fried L.P. (2008). Initial manifestations of frailty criteria and the development of frailty phenotype in the Women’s Health and Aging Study II. J. Gerontol. Ser. A.

[4] Ishii K., Ogawa W., Kimura Y., Kusakabe T., Miyazaki R., Sanada K., Satoh-Asahara N., Someya Y., Tamura Y., Ueki K. (2024). Diagnosis of sarcopenic obesity in Japan: Consensus statement of the Japanese Working Group on Sarcopenic Obesity. Geriatr. Gerontol. Int..

[5] Wei S., Nguyen T.T., Zhang Y., Ryu D., Gariani K. (2023). Sarcopenic obesity: Epidemiology, pathophysiology, cardiovascular disease, mortality, and management. Front. Endocrinol..

[6] Shiraishi A., Yoshimura Y., Wakabayashi H., Nagano F., Matsumoto A., Shimazu S., Kido Y., Bise T., Kuzuhara A., Hori K. (2025). Impaired oral status is associated with sarcopenic obesity in post-stroke patients. Gerodontology.

[7] Shirotori S., Hasegawa Y., Nagai K., Kusunoki H., Yoshimura S., Tokumoto K., Hattori H., Tamaki K., Hori K., Kishimoto H. (2025). Is oral function associated with the development of sarcopenic obesity and sarcopenia in older adults? A prospective cohort study. Diseases.

[8] Tanaka T., Hirano H., Ikebe K., Ueda T., Iwasaki M., Minakuchi S., Arai H., Akishita M., Kozaki K., Iijima K. (2024). Consensus statement on “Oral frailty” from the Japan Geriatrics Society, the Japanese Society of Gerodontology, and the Japanese Association on Sarcopenia and Frailty. Geriatr. Gerontol. Int..

[9] Shiota C., Kusama T., Takeuchi K., Kiuchi S., Osaka K. (2023). Oral Hypofunction and Risk of Weight Change among Independent Older Adults. Nutrients.

[10] Tanaka T., Takahashi K., Akishita M., Tsuji T., Iijima K. (2018). Yubi-wakka [finger-ring] test: A practical self-screening method for sarcopenia, and a predictor of disability and mortality among Japanese community-dwelling older adults. Geriatr. Gerontol. Int..

[11] Nokubi T., Yoshimuta Y., Nokubi F., Yasui S., Kusunoki C., Ono T., Maeda Y., Yokota K. (2013). Validity and reliability of a visual scoring method for masticatory ability using test gummy jelly. Gerodontology.

[12] Charlson M.E., Pompei P., Ales K.L., MacKenzie C.R. (1987). A new method of classifying prognostic comorbidity in longitudinal studies: Development and validation. J. Chronic Dis..

[13] Ministry of Health, Labour and Welfare International Statistical Classification of Diseases, Injuries and Causes of Death (Based on ICD-10). https://www.mhlw.go.jp/toukei/sippei/.

[14] Japan Society for the Study of Obesity (2022). Guidelines for the Management of Obesity Disease 2022.

[15] Nakatsu N., Sawa R., Misu S., Ueda Y., Ono R. (2015). Reliability and validity of the Japanese version of the simplified nutritional appetite questionnaire in community-dwelling older adults. Geriatr. Gerontol. Int..

[16] Drewnowski A., Henderson S.A., Driscoll A., Rolls B.J. (1997). The Dietary Variety Score: Assessing diet quality in healthy young and older adults. J. Am. Diet. Assoc..

[17] Arai E., Watanabe Y., Nakagawa S., Ohara Y., Iwasaki M., Hirano H., Ikebe K., Ono T., Iijima K., Adachi A. (2025). Association of oral frailty with medical expenditure in older Japanese adults: The study of late-stage older adults in Tottori (START Tottori). Gerodontology.

[18] Nakagawa S., Miura K., Arai E., Taira K., Watanabe Y., Shirobe M., Motokawa K., Ohara Y., Iwasaki M., Hirano H. (2024). Oral frailty, appetite and dietary variety in late-stage older adults: A cross-sectional study (the STudy of lAte-stage oldeR adulTs in Tottori; START Tottori). Geriatr. Gerontol. Int..

[19] Tun T.Z., Thwin K.M., Takehara S., Ogawa H. (2024). Oral diadochokinesis and potential associated factors in Japanese older adult outpatients. Oral Health Prev. Dent..

[20] Tottori Prefecture Wide-Area Association of Medical Care for the Older Senior Citizens Number of Insured Persons by Municipality. Number of Insured Persons, Tottori Prefecture Wide-Area Association of Medical Care for the Older Senior Citizens. http://www.koureikouiki-tottori.jp/files/original/20230119105509119.pdf.

[21] Tanaka T., Takahashi K., Hirano H., Kikutani T., Watanabe Y., Ohara Y., Furuya H., Tetsuo T., Akishita M., Iijima K. (2018). Oral frailty as a risk factor for physical frailty and mortality in community-dwelling elderly. J. Gerontol. Ser. A.

[22] Barone B., Napolitano L., Abate M., Cirillo L., Reccia P., Passaro F., Turco C., Morra S., Mastrangelo F., Scarpato A. (2022). The role of testosterone in the elderly: What do we know?. Int. J. Mol. Sci..

[23] Fragala M.S., Cadore E.L., Dorgo S., Izquierdo M., Kraemer W.J., Peterson M.D., Ryan E.D. (2019). Resistance training for older adults: Position statement from the National Strength and Conditioning Association. J. Strength Cond. Res..

[24] Grgic J., Garofolini A., Orazem J., Sabol F., Schoenfeld B.J., Pedisic Z. (2020). Effects of resistance training on muscle size and strength in very elderly adults: A systematic review and meta-analysis of randomized controlled trials. Sports Med..

[25] Zouhal H., Jayavel A., Parasuraman K., Hayes L.D., Tourny C., Rhibi F., Laher I., Abderrahman A.B., Hackney A.C. (2022). Effects of exercise training on anabolic and catabolic hormones with advanced age: A systematic review. Sports Med..

[26] Chou Y.F., Banda K.J., Chen R., Sung C.M., Chiang K.J., Chang L.F., Su P.Y., Chou K.R. (2025). Effects of synergistic tongue and chin resistance training on swallowing function, oral intake, and cognitive function in community-dwelling elderly individuals with frailty: A double-blind randomised controlled trial. J. Glob. Health.

[27] Wei J., Zhao Q., Huang W., Liu X., Zhang X. (2024). Analysis of the Occurrence and Influencing Factors of Oral Frailty in Elderly Residents of Elderly Care Facilities. Sichuan Da Xue Xue Bao Yi Xue Ban.

[28] Kumagai S., Watanabe S., Shibata H., Amano H., Fujiwara Y., Shinkai S., Yoshida H., Suzuki T., Yukawa H., Yasumura S. (2003). Effects of dietary variety on declines in high-level functional capacity in elderly people living in a community. Nihon Koshu Eisei Zasshi.

[29] Embling R., Price M.J., Lee M.D., Jones A., Wilkinson L.L. (2023). Associations between dietary variety, portion size and body weight: Prospective evidence from UK Biobank participants. Br. J. Nutr..

[30] Amasene M., Medrano M., Echeverria I., Urquiza M., Rodriguez-Larrad A., Diez A., Labayen I., Ariadna B.B. (2022). Malnutrition and poor physical function are associated with higher comorbidity index in hospitalized older adults. Front. Nutr..

[31] Ministry of Health, Labour and Welfare (2025). 2024.10. Report of the Expert Working Group on the Dietary Reference Intakes for Japanese. https://www.mhlw.go.jp/content/10904750/001316585.pdf.

[32] Kito N., Matsuo K., Ogawa K., Izumi A., Kishima M., Itoda M., Masuda Y. (2019). Positive effects of “textured lunches” gatherings and oral exercises combined with physical exercises on oral and physical function in older individuals: A cluster randomized controlled trial. J. Nutr. Health Aging.

[33] Matsuo K., Kito N., Ogawa K., Izumi A., Masuda Y. (2020). Effects of textured foods on masticatory muscle activity in older adults with oral hypofunction. J. Oral Rehabil..

[34] Hamada Y., Hayashi N. (2021). Chewing increases postprandial diet-induced thermogenesis. Sci. Rep..

[35] Song H.J., Hwang J., Pi S., Ahn S., Heo Y., Park S., Kwon J.W. (2018). The impact of obesity and overweight on medical expenditures and disease incidence in Korea from 2002 to 2013. PLoS ONE.

[36] Slater N., White S., Venables R., Frisher M. (2018). Factors associated with polypharmacy in primary care: A cross-sectional analysis of data from the English Longitudinal Study of Ageing (ELSA). BMJ Open.

[37] Ministry of Health, Labour and Welfare (2024). Publication of the Results of the 2023 National Health and Nutrition Survey. https://www.mhlw.go.jp/stf/newpage_45540.html.

[38] Barazzoni R., Jensen G.L., Correia M.I.T.D., Gonzalez M.C., Higashiguchi T., Shi H.P., Bischoff S.C., Boirie Y., Carrasco F., Cruz-Jentoft A. (2022). Guidance for assessment of the muscle mass phenotypic criterion for the Global Leadership Initiative on Malnutrition (GLIM) diagnosis of malnutrition. Clin. Nutr..

[39] Donini L.M., Busetto L., Bischoff S.C., Cederholm T., Ballesteros-Pomar M.D., Batsis J.A., Bauer J.M., Boirie Y., Cruz-Jentoft A.J., Dicker D. (2022). Definition and diagnostic criteria for sarcopenic obesity: ESPEN and EASO consensus statement. Obes. Facts.

[40] Fujii H., Kodani E., Kaneko T., Nakamura H., Sasabe H., Tamura Y. (2022). Sarcopenia and coexistent risk factors detected using the “Yubi-wakka” (finger-ring) test in adults aged over 65 years in the public annual health check-up in Tama City, Tokyo: A cross-sectional study. BMJ Open.

[41] Komai S., Watanabe Y., Fujiwara Y., Kim H., Edahiro A., Kawai H., Yoshida H., Obuchi S., Tanaka Y., Hirano H. (2016). Association between the nutritional status and the severity of sarcopenia among community-dwelling elderly Japanese people. Nihon Ronen Igakkai Zasshi.

[42] Yamamoto K., Tsuji T., Yamasaki K., Momoki C., Yasui Y., Habu D. (2020). Scoring methods used in the dietary variety score survey to predict malnutrition among older patients receiving home care. Int. J. Older People Nurs..

