# Supplementary material for: Association Between Oral Frailty and Body Mass Index in Late-Stage Older Adults Stratified by Sarcopenia Status
Source: Nutrients. 2026 Aug 28;18(17):2835. doi: 10.3390/nu18172835 (PMC13566916; doi:10.3390/nu18172835)
Supplement: Supplementary file 1 [file nutrients-18-02835-s001.zip › nutrients-4470094-supplementary.pdf]

Supplementary Materials:
Association between oral frailty and body mass index in late-stage older adults stratified by sarcopenia status

Table S1. Multicollinearity statistics

	Sarcopenia risk group		Non-sarcopenia group	
	Tolerance statistics	Variance inflation factor	Tolerance statistics	Variance inflation factor
Age (years)	0.924	1.082	0.904	1.106
Number of medications taken per day	0.942	1.062	0.931	1.074
Simplified Nutritional Appetite Questionnaire	0.969	1.032	0.942	1.062
Dietary Variety Score	0.955	1.047	0.914	1.094
Charlson Comorbidity Index	0.959	1.042	0.959	1.043
Sex	0.932	1.073	0.919	1.088
Oral frailty assessment results	0.918	1.089	0.907	1.103

Notes: All variables included in the multinomial logistic regression model had variance inflation factor values of <5, indicating no multicollinearity.

Table S2. Crude estimates from multinomial logistic regression analysis of factors associated with underweight and obesity.

	**Sarcopenia risk group**				**Non-sarcopenia group**			
	**Normal weight (reference)**				**Normal weight (reference)**			
	**Underweight**		**Obesity**		**Underweight**		**Obesity**	
	**OR (95% CI)**	**P-value**	**OR (95% CI)**	**P-value**	**OR (95% CI)**	**P-value**	**OR (95% CI)**	**P-value**
Age (years)	1.036 (1.005–1.068)	0.021	0.981 (0.935–1.030)	0.448	1.075 (1.031–1.121)	< 0.001	1.004 (0.982–1.027)	0.705

Number of medications taken per day	1.034 (0.985–1.086)	0.176	1.048 (0.975–1.126)	0.207	0.986 (0.915–1.062)	0.706	1.094 (1.060–1.128)	＜0.001
Simplified Nutritional Appetite Questionnaire	0.920 (0.850–0.995)	0.037	1.036 (0.914–1.173)	0.581	0.966 (0.859–1.087)	0.567	1.071 (1.010–1.135)	0.021
Dietary Variety Score	1.072 (1.010–1.138)	0.023	1.043 (0.955–1.139)	0.346	1.064 (0.973–1.163)	0.176	0.994 (0.956–1.034)	0.775
Charlson Comorbidity Index	1.164 (0.993–1.363)	0.060	1.013 (0.786–1.305)	0.923	0.972 (0.768–1.231)	0.815	0.896 (0.798–1.005)	0.062
Sex, male	(reference)		(reference)		(reference)		(reference)	
female	1.276 (0.954–1.707)	0.101	0.640 (0.421–0.974)	0.037	2.647 (1.610–4.352)	＜ 0.001	0.990 (0.819–1.197)	0.918
Oral frailty assessment results, not applicable	(reference)		(reference)		(reference)		(reference)	
applicable	1.487 (1.123–1.970)	0.006	1.329 (0.874–2.021)	0.184	1.157 (0.778–1.720)	0.472	1.183 (0.983–1.425)	0.076

Abbreviations: CI, confidence interval; OR, odds ratio
